# Calciphylaxis following kidney transplantation: a case report

**DOI:** 10.1186/1752-1947-3-9297

**Published:** 2009-11-24

**Authors:** Rajesh Hanvesakul, Michael A Silva, Rahul Hejmadi, Steve Mellor, Andrew R Ready, Paul Cockwell, Nicholas Inston

**Affiliations:** 1 Department of Nephrology, University Hospital Birmingham, Birmingham B15 2TH, UK; 2 Department of Pathology, University Hospital Birmingham, Birmingham B15 2TH, UK

## Abstract

**Introduction:**

Calciphylaxis occurring after kidney transplantation is rare and rarely reported. It results in chronic non-healing wounds and is associated with a poor prognosis and is often fatal. We present a case of proximal lower limb calciphylaxis that occurred early after kidney transplantation. The patient had no classic associated risk factors. He had previously had a total parathyroidectomy but had normal serum calcium-phosphate product and parathyroid hormone levels. The clinical outcome of this case was favorable and highlights some fundamental issues relating to management.

**Case presentation:**

A 70-year-old British Caucasian man with end-stage renal failure secondary to IgA nephropathy presented six months post kidney transplantation with cutaneous calciphylaxis lesions involving the medial aspect of the thigh bilaterally.

**Conclusion:**

To the best of our knowledge, this is the first reported case of rapid onset cutaneous calciphylaxis occurring soon after kidney transplantation that was associated with a favorable outcome. Cutaneous calciphylaxis lesions should be promptly managed with meticulous wound care, antimicrobial therapy and the correction of calcium-phosphate product where indicated.

## Introduction

Calciphylaxis or calcific uraemic arteriolopathy (CUA) is a disease that involves calcium deposition in the walls of small- and medium-sized arteries with consequent ischaemic necrosis and gangrene of the supplying tissue. This condition was first described in the dialysis population [1] and contrary to its name, is not exclusive to uraemic patients but may occur in nonuraemic conditions such as primary hyperparathyroidism, malignancy, alcoholic liver disease, connective tissue disease, diabetes mellitus, Crohn's disease and corticosteroid use [2]. CUA has a slow and indolent onset that classically presents in the distal lower limbs. However, it may occur in any part of the body, including visceral tissue [3,4]. In general, systemic calciphylaxis has a poor prognosis and is often fatal. The pathogenesis of this disease is not well understood but may involve abnormal calcium phosphate homeostasis. There are also several recognized risk factors associated with the development of CUA, such as hyperparathyroidism, elevated calcium-phosphate product (multiplying serum calcium and phosphate values [normal range less than 4.5]), diabetes mellitus, obesity, coagulopathies, warfarin or iron dextran treatment.

There have been a few reported cases of CUA occurring in patients late after kidney transplantation [4-6]. We describe an unusual case of rapid onset CUA with superimposed infection in a patient six months after kidney transplantation but with normal serum calcium levels despite having had a total parathyroidectomy.

## Case presentation

A 70-year-old British Caucasian man with end stage renal failure (ESRF) secondary to IgA nephropathy received a deceased donor kidney transplant on the 14th January 2008. He had been diagnosed with ESRF 15 years previously, and was initially dialysing via peritoneal dialysis and later via haemodialysis. At the time of transplant, his only remaining vascular access was a long-term intravenous catheter. Many years previously he had undergone a total parathyroidectomy for tertiary hyperparathyroidism and therefore took regular calcium supplements and alfacalcidol at a dose of 0.25 μg/day. Both pre- and post-kidney transplant parathyroid hormone (PTH) and serum calcium levels were within normal ranges with a calcium-phosphate product of less than 4. Post-transplant recovery was complicated by delayed graft function requiring dialysis for 14 days, transplant wound dehiscence requiring V.A.C.^® ^dressing, and bladder outflow obstruction requiring intermittent self-catheterization. Despite these complications, he continued to improve with a good urine output and a glomerular filtration rate of 38.9 ml/min and was discharged home.

**Figure 1:**
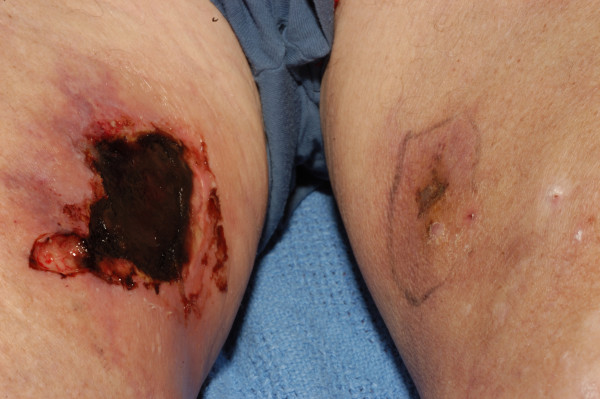
**On the right leg, a well-demarcated and gangrenous lesion is characteristic of calciphylaxis**. In comparison, the lesions on the left leg are those in the early changes of calciphylaxis.

**Figure 2:**
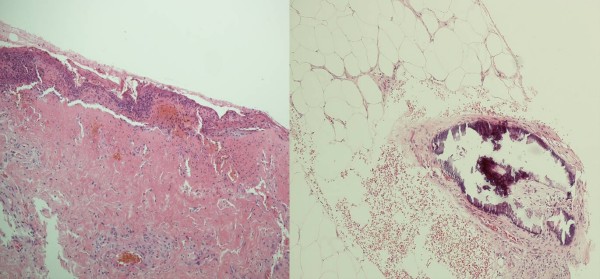
**Histology showing ischaemic skin changes with ulceration, inflammation, scarring (right) and calcification in the medium-sized arteries in the subcutaneous fat (left), typical of calciphylaxis (H & E, ×200)**.

During routine follow-up at the outpatient clinic, the patient's calcium supplements were modified. He was admitted four months later with a complaint of sudden onset of painless lesions bilaterally on the medial aspect of his thighs. They appeared within the 48 hours prior to admission and were not associated with a history of trauma. Serum levels of calcium, phosphate, PTH and calcium-phosphate product were found to be normal. Clinically, both lesions were well demarcated, gangrenous in appearance and foul smelling (Figure 1). Peripheral pulses were intact. Swabs taken from this lesion grew *Pseudomonas *and coliforms sensitive to meropenem. A wedge biopsy of the lesion confirmed the diagnosis of calciphylaxis (Figure 2). Intravenous antibiotic was immediately introduced and all calcium supplements were stopped. Over a period of one month, the lesions had fully resolved.

## Conclusion

We describe an unusual presentation of proximal lower limb calciphylaxis with superimposed infection. Interestingly, this patient did not have any of the recognized risk factors associated with the development of CUA. The clinical outcome in this case has been favorable and emphasizes the importance of early diagnosis, aggressive antimicrobial therapy and wound management. CUA may present as mild erythematous patches, livido reticularis, painful nodules or necrotic ulcerating lesions, giving various possible differential diagnoses. Gold standard treatment for CUA includes diagnostic biopsy to refute other causes of cutaneous lesions, treatment of hyperparathyroidism, reduction of calcium-phosphate product, anti-microbial therapy and meticulous wound care. Several therapeutic strategies for the treatment of CUA have been tested including parathyroidectomy, hyperbaric oxygen therapy, sodium thiosulfate infusion, tissue plasminogen activator and bisphosphonates but none have shown any consistent benefit [7]. Cinacalcet has been used in the treatment of CUA in individuals who were anaesthetically unfit for parathyroidectomy. It inhibits PTH release and has been shown to be effective in the treatment of CUA in cases where PTH levels were found to be high (secondary hyperparathyroidism) [8,9]. In general, CUA is associated with a poor prognosis if not aggressively managed.

## List of abbreviations

CUA: calcific uraemic arteriolopathy; ESRF: end stage renal failure; GFR: glomerular filtration rate; PTH: parathyroid hormone.

## Competing interests

The authors declare that they have no competing interests.

## Authors' contributions

RH, MAS, SM, ARR, PC and NI all made substantial intellectual contributions to the preparation of the manuscript. RHe performed the histological examination of the lesion. All authors read and approved the final manuscript.

## Consent

Written informed consent was obtained from the patient for publication of this case report and accompanying images. A copy of the written consent is available for review by the Editor-in-Chief of this journal.
